# Neutrophil to Lymphocyte Ratio and Spontaneous Bacterial Peritonitis among Cirrhotic Patients: A Systematic Review and Meta-analysis

**DOI:** 10.1155/2022/8604060

**Published:** 2022-09-27

**Authors:** Seyed Arsalan Seyedi, Seyed Ali Nabipoorashrafi, Jairo Hernandez, Andrew Nguyen, Brandon Lucke-Wold, Shima Nourigheimasi, Shokoufeh Khanzadeh

**Affiliations:** ^1^Endocrinology and Metabolism Research Center (EMRC), Vali-Asr Hospital, School of Medicine, Tehran, Iran; ^2^Department of Neurosurgery, University of Florida, Gainesville, USA; ^3^School of Medicine, Arak University of Medical Sciences, Arak, Iran; ^4^Student Research Committee, Tabriz University of Medical Sciences, Tabriz, Iran

## Abstract

**Background:**

The goal of this systematic review and meta-analysis was analyzing published studies on the role of neutrophil to lymphocyte ratio (NLR) in infection and spatially spontaneous bacterial peritonitis (SBP) among cirrhotic patients.

**Methods:**

PubMed, Web of Science, and Scopus were searched until May 24, 2022. The Newcastle–Ottawa scale was used for quality assessment.

**Results:**

Of 14 studies included in our study, six studies were on infection with 2786 hospitalized cirrhotic patients, of whom 934 developed an infection. Other studies were on SBP with 1573 cirrhotic patients with ascites, of whom 557 developed SBP. The pooled results showed that there was no difference in NLR levels between hospitalized cirrhotic patients who developed infection compared to those who did not (random-effects model: SMD = 0.63, 95% CI = −0.01–1.27, *p*=0.054). However, cirrhotic patients with ascites who developed SBP had elevated levels of NLR compared to those who did not (random-effects model: SMD = 1.05, 95% CI = 0.52–1.57,*p* < 0.001). This difference remained significant in prospective studies (SMD = 0.94, 95% CI = 0.51–1.38,*p* < 0.001) but not in retrospective studies (SMD = 1.37, 95% CI = −0.56–3.29,*p*=0.165), in the subgroup analysis according to the study design. The pooled sensitivity of NLR was 92.07% (95% CI = 74.85%–97.84%) and the pooled specificity was 72.58% (95% CI = 57.72%–83.69%). The pooled positive likelihood ratio, negative likelihood ratio, DOR of NLR were 3.35(95%CI = 2.06–5.46), 0.10 (95%CI = 0.03–0.38), and 30.78 (95%CI = 7.01–135.04), respectively.

**Conclusion:**

Our results support NLR to be a valid biomarker that can be readily integrated into clinical settings to help in the prevention and prediction of SBP among cirrhotic patients.

## 1. Background

The role of the neutrophil to lymphocyte ratio (NLR) has been extensively studied to determine its diagnostic and prognostic utility within various pathologies. This has included detecting the presence and clinical progression of diseases such as cardiac failure [1]. The NLR is speculated to give practical insight into the immune system activity with irregular ranges signaling disease severity or imminent infection [2, 3]. Assessing the diagnostic value of NLR in targeting liver disease has gained particular interest, namely, in those with nonalcoholic fatty liver disease (NAFLD), hepatocellular carcinoma (HCC), and others [4–6]. Liver disease patients are commonly observed with cirrhosis, which indicates their disease has progressed into physical scarring of the tissue [7]. An advanced yet common complication of the cirrhosis population is spontaneous bacterial peritonitis (SBP), in which the ascitic fluid of the abdomen carries infectious bacteria. There are no clear sources of infection for SBP within the abdomen, the defining criterion of its etymology. [8] The progression of hospitalized patients afflicted with SBP is particularly concerned with an in-hospital mortality rate of approximately 17% [9]. Therefore, the sequelae of SBP highlight the increasing value in establishing reliable biomarkers for diagnostic application. To our knowledge, there exists no systematic review of the current research regarding the diagnostic utility of NLR in infection or SBP. This systematic review and meta-analysis stand as a coherent synthesis of the current literature regarding liver cirrhosis infections to guide clinical decision-making with NLR.

## 2. Methods

### 2.1. Eligibility Criteria

We included human studies according to the following eligibility criteria based on PICO.

- Population: cirrhotic patients who developed an infection. If a study reported only SBP among these patients, it would be included in the separate analysis concerning SBP cases solely. This action was taken to increase the homogeneity between studies.
- Intervention. NLR
- Control. Cirrhotic patients who did not develop any types of infection
- Outcomes. The prognostic performance of NLR
- Study design. We expected papers to be case-control or cross-sectional. However, we did not limit our search to any particular research design.

### 2.2. Search Strategy and Study Selection

We performed a comprehensive literature search in the databases of PubMed, Web of Science, and Scopus, from inception until May 24, 2022, based on the following search strategy: (“neutrophil to lymphocyte ratio” OR NLR) AND (Cirrhosis OR (Liver AND (Disease OR Failure))) AND (Infection *∗* OR “spontaneous bacterial peritonitis”).

Two authors independently screened abstracts. Full text of relative papers was retrieved. We also investigated the references of relevant reviews or original articles in order to identify further eligible studies. A third person resolved disagreements between the two authors who screened the papers.

### 2.3. Data Extract and Quality Assessment

We extracted the following data: the first author, year of publication, study location, the number of cases and controls separately, mean ± SD of NLR level in cases and controls, or sufficient data for estimating the mean ± SD such as median and interquartile range(IQR) or/and range. The Newcastle–Ottawa Scale (NOS) [10] was applied to evaluate the studies' quality. This scale assessed three elements: selection of the cohort, comparability of cohorts based on the design or analysis, how the exposure was ascertained, and how outcomes of interest were evaluated. The high quality was considered as achieving six or more stars.

### 2.4. Statistical Analysis

Standardized mean differences (SMD) and 95% confidence intervals (CIs), were applied to report forest plots of continuous data. In addition, subgroup analysis was performed according to the study design. We considered *p* < 0.05 as statistically significant. If a study did not report mean ± SD, we estimated them from the median and range [11].

We used *Q* statistic (significance level at *p* < 0.10) to test the heterogeneity of SMD across included articles. Additionally, we calculated the I^2^ statistic as a quantitative measurement to evaluate inconsistency across studies (I^2^ < 25%, no heterogeneity; I^2^ between 25% and 50%, moderate heterogeneity; I^2^ between 50% and 75%, large heterogeneity; and I^2^ > 75%, extreme heterogeneity). Because of high heterogeneity, we applied a random-effects model to report the pooled SMD and corresponding 95% confidence intervals.

We used the Begg and Egger test to assess the potential publication bias (at the *p* < 0.05 level of significance). In order to evaluate the diagnostic value of NLR, we used the “metandi” command to report the summary receiver operating characteristic (SROC) curve, the sensitivity, specificity, diagnostic odds ratio (DOR), negative likelihood ratio, and positive likelihood ratio. We used Stata 14 (STATA Corp., College Station, TX, USA) for the statistical analyses. The current study completely followed the PRISMA statement about the reporting of systematic reviews and meta-analyses [12] and the broader EQUATOR guidelines [13].

## 3. Results

### 3.1. Search and Selection of Literature

**Figure 1:**
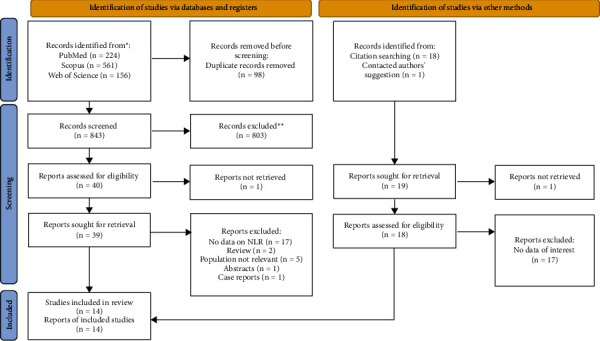
PRISMA 2020 flow diagram for new systematic reviews including searches of databases, registers, and other sources.

A total of 960 records were retrieved in the database search and manual search of the citation list of articles. After the exclusion of duplicates and not relevant records, 14 studies were included in the systematic review and meta-analysis [14–27]. Of 14 studies, six studies were on infection with 2786 hospitalized cirrhotic patients, of whom 934 developed infection [18, 20–22, 24, 26]. Others were on SBP with 1573 cirrhotic patients with ascites, of whom 557 developed SBP [14–17, 19, 23, 25, 27]. The process of inclusion and exclusion is detailed in the PRISMA flow diagram, which is provided in Figure 1.

### 3.2. Characteristics of Included Studies

The characteristics and methodological qualities of these studies are shown in Table 1. The overall study quality ranged from 6 to 8 stars. Fourteen studies were included in our systematic review and meta-analysis [14–27]. Seven studies were prospective [15–17, 19, 20, 23, 25] and others were retrospective [14, 18, 21, 22, 24, 26, 27]. All of them were written in English. Seven studies reported the results of receiver operating characteristic (ROC) curve analysis, including the best cutoff point, sensitivity, and specificity of NLR in the prediction of SBP [14–17, 19, 24, 27].

### 3.3. Association of the NLR and Infection among Hospitalized Cirrhotic Patients

**Figure 2:**
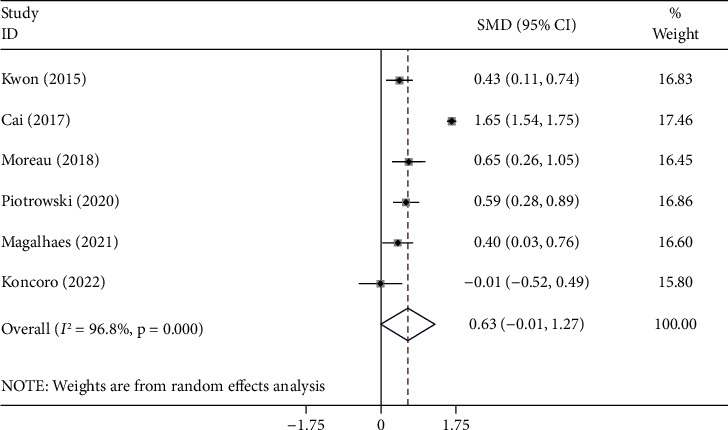
Meta-analysis of differences in the NLR level between hospitalized cirrhotic patients who developed infection compared to those who did not.

The random-effects model was applied to the pooled meta-analysis, as statistical heterogeneity existed among studies (I^2^ = 96.8%, *p* < 0.001). The pooled results showed that there was no difference in NLR levels between hospitalized cirrhotic patients who developed infection compared to those who did not (SMD = 0.63, 95% CI = -0.01–1.27, *p*=0.054) (Figure 2).

**Figure 3:**
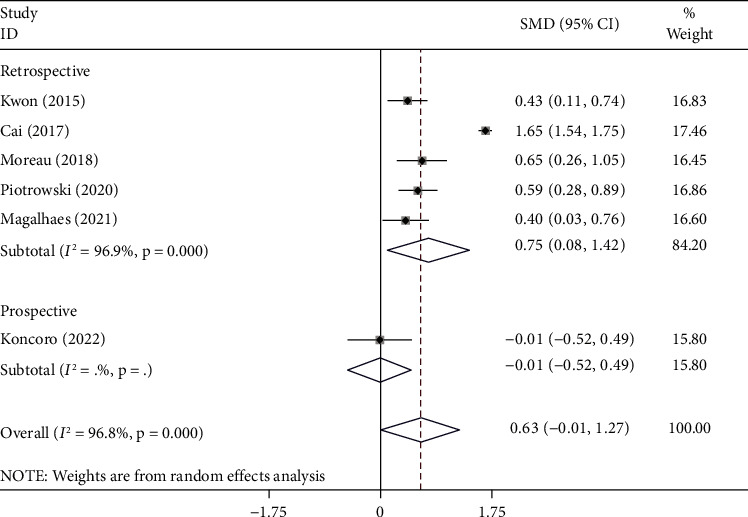
Subgroup analysis of differences in the NLR level between hospitalized cirrhotic patients who developed infection compared to those who did not, according to the study design.

Figure 3 shows the subgroup analysis according to the study design. We found that hospitalized cirrhotic patients who developed infection had elevated levels of NLR compared to those who did not in retrospective studies (SMD = 0.75, 95% CI = 0.08–1.42, *p*=0.028) but not in one prospective study (SMD = −0.01, 95% CI = −0.52–0.49, *p*=0.965).

### 3.4. Association of the NLR and SBP among Cirrhotic Patients with Ascites

**Figure 4:**
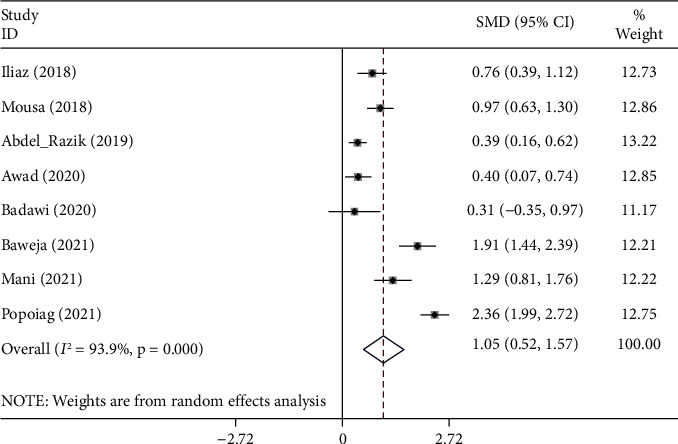
Meta-analysis of differences in the NLR level between cirrhotic patients with ascites who developed SBP had elevated levels of NLR compared to those who did not.

The random-effects model was applied to the pooled meta-analysis, as statistical heterogeneity existed among studies (I^2^ = 93.9%, *p* < 0.001). The pooled results indicated that cirrhotic patients with ascites who developed SBP had elevated levels of NLR compared to those who did not (SMD = 1.05, 95% CI = 0.52–1.57, *p* < 0.001) (Figure 4).

**Figure 5:**
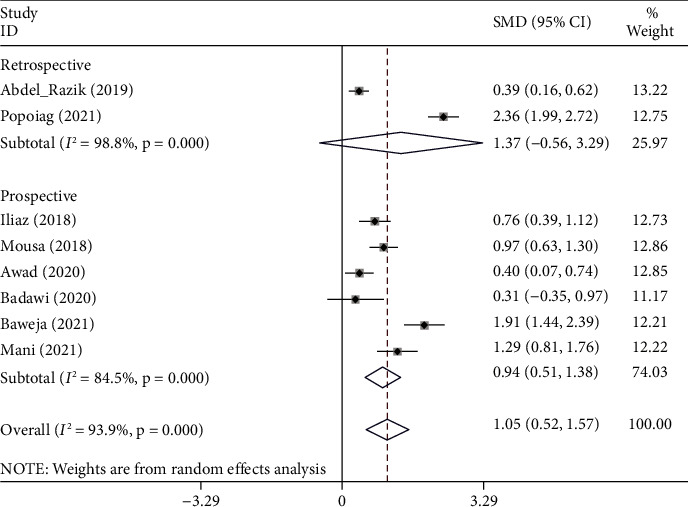
Subgroup analysis of differences in the NLR level between cirrhotic patients with ascites who developed SBP had elevated levels of the NLR compared to those who did not, according to the study design.

In subgroup analysis according to the study design, cirrhotic patients with ascites who developed SBP had elevated levels of NLR compared to those who did not in prospective studies (SMD = 0.94, 95% CI = 0.51–1.38, *p* < 0.001) but not in retrospective studies (SMD = 1.37, 95% CI = -0.56–3.29, *p*=0.165) (Figure 5).

### 3.5. Diagnostic Value of the NLR in SBP

**Figure 6:**
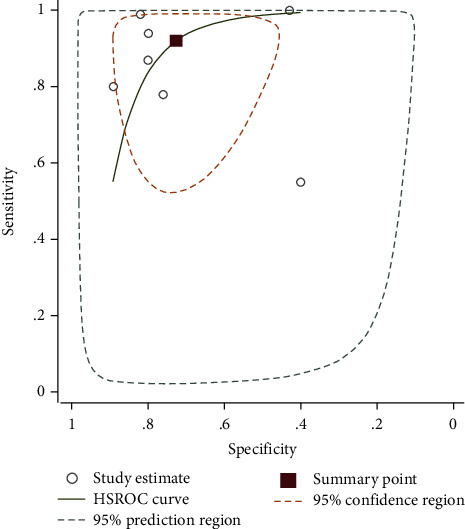
SROC curve included studies in the meta-analysis of the association of the NLR with SBP.

The pooled sensitivity of seven studies was 92.07% (95% CI = 74.85%–97.84%), and the pooled specificity was 72.58% (95% CI = 57.72%–83.69%). The pooled positive likelihood ratio, negative likelihood ratio, DOR of NLR were 3.35(95%CI = 2.06–5.46), 0.10 (95%CI = 0.03–0.38), and 30.78 (95%CI = 7.01–135.04), respectively (Figure 6).

### 3.6. Publication Bias

**Figure 7:**
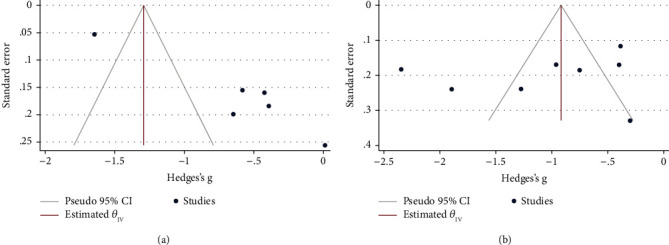
Funnel plots assessing publication bias among studies; (a) Studies on infection; (b) Studies on SBP.

As seen in Figure 7, there was some indication of publication bias among studies on the usefulness of NLR for predicting infection (Egger's test *p* < 0.001). However, studies on the usefulness of NLR for predicting SBP had no publication bias (Egger's test *p*=0.90).

## 4. Discussion

Our study explored infection prevalence in two groups of individuals with cirrhosis and ascites-afflicted cirrhosis. Only the latter investigation revealed an increase in NLR amongst cirrhotic patients with infection (*p* < 0.001). Previous studies have assessed the utility of other parameters in SBP diagnosis, displaying comparable detection capacity. In a cohort of 7299, higher proton-pump inhibitor (PPI) concentration was associated with a greater likelihood of SBP (Odds ratio(OR) = 1.75). The prescription of PPIs can be used to mitigate the volume of gastric acid elevated by cirrhosis. As an expense, the fluctuation of pH levels can alter the composition of the endogenous gut flora, encouraging infection [28–30]. Other inflammatory biomarkers, including macrophage inflammatory protein-1 beta (MIP-1B), have displayed sensitivities and specificities ranging from 72.7–80% and 76.1%–100%, respectively [31, 32]. Of the included studies in our meta-analysis, 7/8 concluded NLR was significantly associated with infection. After the meta-analysis, we found no significant increase in NLR levels in hospitalized cirrhotic patients who developed an infection. An important consideration, however, was the variation in sensitivity, specificities, and cutoff values at which NLR could be used as a diagnostic marker. This heterogeneity prompts further exploration of NLR regarding cutoff guidelines and more to be effective in an environment without controls.

Additionally, two of these included studies further assessed the prognostic capacity of NLR, both finding differences in SBP patients with and without mortality [19, 23]. Illiaz and colleagues demonstrated that the NLR was useful in predicting 30-day and 3-month mortality. Those with mortality displayed mean NLR levels of 16.5 ± 11.8, and without mortality, 7.8 ± 9 (*p*=0.002). Interestingly, they also noted a difference in the symptoms of ascites-afflicted cirrhosis patients with and without SBP (*p* < 0.001). This demonstrates the varying presentations of SBP, with the consideration that SBP can manifest asymptomatically. Upon controlling for symptom prevalence within mortality and nonmortality SBP-affected patients, there was no change in the significant association noted.

Understanding the immunological cascade catalyzed by infection in the context of cirrhosis is critical for the synthesis of previous literature and the present study's results. During infection, neutrophils carry a vital defensive role through tasks such as phagocytosis, degranulation, and release of reactive oxygen species (ROS) [33–36]. This is preceded by intravascular migration via chemokine gradient signaling and extravasation to the focused site. A suspected consequence of cirrhosis, with or without infection, is the increased rate of bacterial translocation, particularly through the intestinal wall. Prior investigations have illustrated the increased presence of proinflammatory and neutrophilic biomarkers in cirrhotic plasma. These have revealed a significant increase in nitrates and proinflammatory cytokines in response to a likewise increased amount of endotoxins [35, 37]. Bacteremia of Gram-negative bacteria typically peaks within a few days, consequentially cleared by neutrophils, and finally marked by aberrant levels of LBS in the following 72 hours [35].

Despite the increased inflammatory activity, complications of cirrhosis lead to the overall potency of WBC being impaired. These effects include a reduction in vital proteins such as toll-like receptors (TLR) and intensified cytopenia, among other defects, furthering susceptibility to infection as a whole [37]. Conversely, cirrhosis is often accompanied by lymphopenia secondary to hypersplenism [38]. The upregulated elimination of white blood cells (WBC) occurs due to the stress from portal hypertension, creating abnormal pressure on the spleen. Additionally, the decrease in lymphocytes may occur as a negative reaction to the upregulated inflammatory response [39]. This may create unequal proportions of neutrophils to lymphocytes, as observed in other pathologies [32].

A subgroup analysis evaluating NLR in cirrhotic patients with ascites developing SBP showed significant prognostic capacity (*p* < 0.001). Ascites is a pathologic accumulation of peritoneal fluid and a very common manifestation of decompensated cirrhosis. Patients with ascites-afflicted cirrhosis have progressed to a stage in which the dysfunction of the liver has allowed for severe alteration of the local hemodynamic balance. This typically indicates a phase in which the complications of cirrhosis are only further exacerbated, including by bacterial translocation, sparing greater opportunity for infective progression [40].

The pathogenesis of ascites remains incompletely understood, but several factors have been identified as contributors. Continued injury to the liver leads to portal hypertension, which causes backflow and the status of vasodilatory substances. These vasodilatory substances lead to hypoperfusion of the renal system, which leads to renin secretion and subsequent aldosterone/vasopressin release, increased thirst drive, and increased renal blood flow [40, 41]. Excessive fluid retention leads to an increase in hydrostatic pressure and the stasis of vasodilatory substances increases vascular wall permeability and concurrently decreases osmotic pressure through absolute or relative hypoalbuminemia. These three parameters, which are described in the Starling equation, overwhelm reabsorption capacity and lead to ascites [42]. Bacterial translocation, which is defined as the migration of bacteria from the intestinal lumen to mesenteric lymph nodes or other extraintestinal sites, represents a disruption of host/flora equilibrium which eventually leads to infection [28]. In cirrhosis and ascites patients, intestinal permeability is significantly elevated, leading to greater bacterial translocation and a higher risk for SBP. Using NLR as a prognostic/diagnostic factor for patients in this state of hemodynamic derangement might be especially helpful since they are significantly more susceptible to infection [43].

The results indicate a difference in NLR SBP predictive value in retrospective (*p*=0.028) compared to prospective studies (*p*=0.965) in hospitalized cirrhotic patients. We speculate that this effect may be due to the limited number of prospective studies compared to retrospective studies. Thus, more prospective studies may help clarify whether there is a difference between retrospective and prospective studies in the context of the NLR and its predictive value for bacterial infection in hospitalized cirrhotic patients. In a subgroup analysis according to the study design, NLR was significantly associated with infection in hospitalized cirrhotic patients with ascites in prospective studies (*p* < 0.001) but not in retrospective studies (*p*=0.165). This could be due to the greater number of prospective studies included in this subgroup analysis compared to the number of retrospective studies. Therefore, more retrospective studies would be useful for evaluating NLR utility in cirrhotic patients with ascites.

Our study has a few limitations that are important to address. The main limitation of this study is the small number of papers that were included in the meta-analysis of the association of NLR with infection. As such, our results may be limited in power, and additional studies would be warranted to further strengthen the results of our study. Furthermore, the studies included in our analysis exhibited high heterogeneity. Although this was accounted for with the random-effect model, such measures may not entirely eliminate the issue of heterogeneity. Nonetheless, our systematic search, in conjunction with a manual review of references from resulting articles, has ensured a thorough and reliable search of the literature and serves as a notable strength of this study.

## 5. Conclusion

In conclusion, the data regarding cirrhotic patients suggest that the NLR may be useful as an independent diagnostic marker of SBP. Further studies need to be conducted to determine precise cutoff guidelines in which to utilize the NLR.

## Figures and Tables

**Table 1:** General characteristics of included studies.

First author	Year	Country	Design	End point	NLR cutoff point	Sensitivity	Specificity	*Cases*		*Controls*		NOS score
								N	NLR	N	NLR	
Kwon [21]	2015	South Korea	Retrospective	Infection	—	—	—	58	8.30 ± 10.10	126	4.90 ± 6.80	7

Cai [18]	2017	China	Retrospective	Infection	—	—	—	708	7.70 ± 5.00	1358	2.49 ± 1.48	8

Iliaz [19]	2018	Turkey	Prospective	SBP	9.2	78%	76%	70	10.20 ± 10.40	55	4.10 ± 3.10	6

Moreau [24]	2018	Belgium	Retrospective	Infection	—	—	—	45	17.33 ± 15.31	63	9.66 ± 8.34	7

Mousa [25]	2018	Egypt	Prospective	SBP	2.89	80%	89%	126	5.46 ± 4.12	54	2.10 ± 0.76	7

Abdel-Razik [14]	2019	Egypt	Retrospective	SBP	2.50	87%	80%	84	2.40 ± 1.80	598	1.90 ± 1.20	7

Awad [15]	2020	Egypt	Prospective	SBP	3.50	100%	43%	70	26.34 ± 72.67	70	5.65 ± 3.80	6

Badawi [16]	2020	Egypt	Prospective	SBP	3.53	55%	40%	22	5.96 ± 6.15	15	4.42 ± 2.37	6

Piotrowski [26]	2020	Poland	Retrospective	Infection	3.96	43%	86%	60	3.30 ± 3.45	149	2.05 ± 1.28	6

Baweja [17]	2021	India	Prospective	SBP	3.38	94%	80%	50	6.73 ± 2.70	50	2.81 ± 1.06	6

Magalhaes [22]	2021	Portugal	Retrospective	Infection	—	—	—	43	4.80 ± 1.92	96	4.00 ± 2.07	7

Mani [23]	2021	Greece	Prospective	SBP	—	—	—	63	0.80 ± 0.09	30	0.68 ± 0.10	6

Popoiag [27]	2021	Romania	Retrospective	SBP	2.40	99%	82%	72	3.67 ± 1.13	14460	1.87 ± 0.49	8

Koncoro [20]	2022	Indonesia	Prospective	Infection	—	—	—	20	7.23 ± 8.46	60	7.36 ± 12.25	6

N:number; NLR: neutrophil to lymphocyte ratio; SBP: spontaneous bacterial peritonitis; and NOS: the Newcastle–Ottawa quality assessment scale.

## Data Availability

The dataset supporting the conclusions of this article is included within the article.

## Abbreviations

N: Number
NLR: Neutrophil to lymphocyte ratio
NAFLD: Nonalcoholic fatty liver disease
HCC: Hepatocellular carcinoma
SBP: Spontaneous bacterial peritonitis
PRISMA: Preferred Reporting Items for Systematic Reviews and Meta-Analyses
NOS: Newcastle–Ottawa Quality Assessment Scale
SMD: Standard mean difference
95% CI: 95% confidence interval
WMD: Weighted mean difference
SROC: Summary receiver operating characteristic
ROC: Receiver operating characteristic
DOR: Diagnostic odds ratio
PPI: Proton-pump inhibitor
MIP-1B: Macrophage inflammatory protein-1 beta
ROS: Reactive-oxygen species
TLR: Toll-like receptors
WBC: White blood cells
CRP: C-reactive protein
AF: Lactate ascitic fluid lactate
ESR: Erythrocyte sedimentation rate.

## References

[1] Bhat T., Teli S., Rijal J. (2013). Neutrophil to lymphocyte ratio and cardiovascular diseases: a review. *Expert Review of Cardiovascular Therapy*.

[2] Lee J. H., Song S., Yoon S. Y., Lim C. S., Song J. W., Kim H. S. (2016). Neutrophil to lymphocyte ratio and platelet to lymphocyte ratio as diagnostic markers for pneumonia severity. *British Journal of Biomedical Science*.

[3] Yu B. Z., Fu J., Chai W., Hao L. B., Chen J. Y. (2020). Neutrophil to lymphocyte ratio as a predictor for diagnosis of early Periprosthetic joint infection. *BMC Musculoskeletal Disorders*.

[4] Absenger G., Szkandera J., Pichler M. (2013). A derived neutrophil to lymphocyte ratio predicts clinical outcome in stage II and III colon cancer patients. *British Journal of Cancer*.

[5] Oh B. S., Jang J. W., Kwon J. H. (2013). Prognostic value of C-reactive protein and neutrophil-to-lymphocyte ratio in patients with hepatocellular carcinoma. *BMC Cancer*.

[6] Alkhouri N., Morris-Stiff G., Campbell C. (2012). Neutrophil to lymphocyte ratio: a new marker for predicting steatohepatitis and fibrosis in patients with nonalcoholic fatty liver disease. *Liver International*.

[7] Schuppan D., Afdhal N. H. (2008). Liver cirrhosis. *The Lancet*.

[8] Garcia M., Sanroman A. L., Gisbert J. P., Martin de Argila C., Moreira V. F. (1992). Aeromonas sobria spontaneous bacterial peritonitis. *American Journal of Gastroenterology*.

[9] Niu B., Kim B., Limketkai B. N. (2018). Mortality from spontaneous bacterial peritonitis among hospitalized patients in the USA. *Digestive Diseases and Sciences*.

[10] Wells G. A., Shea B., O’Connell D. (2000). *The Newcastle-Ottawa Scale (NOS) for Assessing the Quality of Nonrandomised Studies in Meta-Analyses*.

[11] Wan X., Wang W., Liu J., Tong T. (2014). Estimating the sample mean and standard deviation from the sample size, median, range and/or interquartile range. *BMC Medical Research Methodology*.

[12] Page M. J., McKenzie J. E., Bossuyt P. M. (2021). Updating guidance for reporting systematic reviews: development of the PRISMA 2020 statement. *Journal of Clinical Epidemiology*.

[13] Altman D. G., Simera I., Hoey J., Moher D., Schulz K. (2008). EQUATOR: reporting guidelines for health research. *Open Medicine: A Peer-Reviewed, Independent, Open-Access Journal*.

[14] Abdel-Razik A., Mousa N., Abdel-Aziz M. (2019). Mansoura simple scoring system for prediction of spontaneous bacterial peritonitis: lesson learnt. *European Journal of Gastroenterology and Hepatology*.

[15] Awad S., Ahmed E., Mohamed E. (2020). Role of combined blood neutrophil-lymphocyte ratio and C-reactive protein in diagnosis of spontaneous bacterial peritonitis. *Benha Journal of Applied Sciences*.

[16] Badawi R., Asghar M. N., Abd-Elsalam S. (2020). Amyloid a in serum and ascitic fluid as a novel diagnostic marker of spontaneous bacterial peritonitis. *Anti-Inflammatory & Anti-Allergy Agents in Medicinal Chemistry*.

[17] Baweja A., Jhamb R., Kumar R., Garg S., Gogoi P. (2021). Clinical utility of neutrophil lymphocyte ratio (nlr) as a marker of spontaneous bacterial peritonitis (sbp) in patients with cirrhosis-an exploratory study. *International Journal of Science and Research Archive*.

[18] Cai Y.-J., Dong J. J., Dong J. Z. (2017). Neutrophil-lymphocyte ratio predicts hospital-acquired bacterial infections in decompensated cirrhosis. *Clinica Chimica Acta*.

[19] Iliaz R., Ozpolat T., Baran B. (2018). Predicting mortality in patients with spontaneous bacterial peritonitis using routine inflammatory and biochemical markers. *European Journal of Gastroenterology and Hepatology*.

[20] Koncoro H., Wibawa D. N. (2017). C-reactive protein and resistin detect bacterial infection in liver cirrhosis. *International Journal of Gastroenterology, Hepatology, Transplant and Nutrition*.

[21] Kwon J. H., Jang J. W., Kim Y. W. (2015). The usefulness of C-reactive protein and neutrophil-to-lymphocyte ratio for predicting the outcome in hospitalized patients with liver cirrhosis. *BMC Gastroenterology*.

[22] Magalhães R. D. S., Magalhaes J., Sousa-Pinto B., Curdia Goncalves T., Rosa B., Cotter J. (2021). Neutrophil-to-lymphocyte ratio: an accurate method for diagnosing infection in cirrhosis. *Postgraduate Medicine*.

[23] Mani I., Alexopoulos T., Hadziyannis E., Tsiriga A., Vourli G., Alexopoulou A. (2021). An exploratory study of ascitic fluid lactate as prognostic factor of mortality in cirrhotic patients with spontaneous bacterial peritonitis. *European Journal of Gastroenterology and Hepatology*.

[24] Moreau N., Wittebole X., Fleury Y., Forget P., Laterre P. F., Castanares-Zapatero D. (2018). Neutrophil-to-lymphocyte ratio predicts death in acute-on-chronic liver failure patients admitted to the intensive care unit: a retrospective cohort study. *Shock*.

[25] Mousa N., Besheer T., Abdel-Razik A. (2018). Can combined blood neutrophil to lymphocyte ratio and C-reactive protein be used for diagnosis of spontaneous bacterial peritonitis?. *British Journal of Biomedical Science*.

[26] Piotrowski D., Saczewska-Piotrowska A., Jaroszewicz J., Boron-Kaczmarska A. (2020). Lymphocyte-to-monocyte ratio as the best simple predictor of bacterial infection in patients with liver cirrhosis. *International Journal of Environmental Research and Public Health*.

[27] Popoiag R.-E., Suceveanu A. I., Suceveanu A. P. (2021). Predictors of spontaneous bacterial peritonitis in Romanian adults with liver cirrhosis: focus on the neutrophil-to-lymphocyte ratio. *Experimental and Therapeutic Medicine*.

[28] Wiest R., Krag A., Gerbes A. (2012). Spontaneous bacterial peritonitis: recent guidelines and beyond. *Gut*.

[29] Ngo H., Gantioque R. (2017). Predictors of spontaneous bacterial peritonitis (SBP) in liver cirrhosis: current knowledge and future frontiers. *Open Journal of Gastroenterology*.

[30] Dam G., Vilstrup H., Watson H., Jepsen P. (2016). Proton pump inhibitors as a risk factor for hepatic encephalopathy and spontaneous bacterial peritonitis in patients with cirrhosis with ascites. *Hepatology*.

[31] Lesinska M., Hartleb M., Gutkowski K., Nowakowska-Dulawa E (2014). Procalcitonin and macrophage inflammatory protein-1 beta (MIP-1*β*) in serum and peritoneal fluid of patients with decompensated cirrhosis and spontaneous bacterial peritonitis. *Advances in Medical Sciences*.

[32] Khorshed S. E., Ibraheem H. A., Awad S. M. (2015). Macrophage inflammatory protein-1 beta (MIP-1<i>*β*</i>) and platelet indices as predictors of spontaneous bacterial Peritonitis<br> —MIP, MPV and PDW in SBP. *Open Journal of Gastroenterology*.

[33] Liu K., Wang FS., Xu R. (2021). Neutrophils in liver diseases: pathogenesis and therapeutic targets. *Cellular and Molecular Immunology*.

[34] Runyon B. A. (2021). *Pathogenesis of Ascites in Patients with Cirrhosis*.

[35] Albillos A., de la Hera A., González M. (2003). Increased lipopolysaccharide binding protein in cirrhotic patients with marked immune and hemodynamic derangement. *Hepatology*.

[36] Thabut D., Massard J., Gangloff A. (2007). Model for end-stage liver disease score and systemic inflammatory response are major prognostic factors in patients with cirrhosis and acute functional renal failure. *Hepatology*.

[37] Dirchwolf M., Ruf A. E. (2015). Role of systemic inflammation in cirrhosis: from pathogenesis to prognosis. *World Journal of Hepatology*.

[38] Qamar A. A., Grace N. D. (2009). Abnormal hematological indices in cirrhosis. *Canadian Journal of Gastroenterology*.

[39] Tuchendler E., Tuchendler P. K., Madej G. (2018). Immunodeficiency caused by cirrhosis. *Clinical and Experimental Hepatology*.

[40] Moore C. M., Van Thiel D. H. (2013). Cirrhotic ascites review: pathophysiology, diagnosis and management. *World Journal of Hepatology*.

[41] Schrier R. W., Arroyo V., Bernardi M., Epstein M., Henriksen J. H., Rodes J. (1988). Peripheral arterial vasodilation hypothesis: a proposal for the initiation of renal sodium and water retention in cirrhosis. *Hepatology*.

[42] Moller S., Henriksen J. H., Bendtsen F. (2008). Pathogenetic background for treatment of ascites and hepatorenal syndrome. *Hepatol Int*.

[43] Li Y. T., Huang J. R., Peng M. L. (2020). Current status and prospects of spontaneous peritonitis in patients with cirrhosis. *BioMed Research International*.

